# Analysis of real-world data demonstrating the efficacy of current management of polycythaemia vera in attaining and maintaining therapeutic haematocrit

**DOI:** 10.1007/s11845-023-03510-7

**Published:** 2023-09-08

**Authors:** Frances D. Buckley, Claire Arnold, Dawn Brass, Mark Catherwood, Mary Frances McMullin

**Affiliations:** 1 Belfast City Hospital, Belfast Trust, Belfast, Northern Ireland; 2 Ninewells Hospital and Medical School, NHS Tayside, Dundee, Scotland; 3 Raigmore Hospital, NHS Highland, Inverness, Scotland; 4 Centre for Medical Education, Queen’s University, Belfast, Northern Ireland

**Keywords:** Haematology, Myeloproliferative neoplasms, Polycythaemia

## Abstract

**Background:**

Polycythaemia vera (PV) is a Philadelphia-negative myeloproliferative neoplasm, typically driven by acquired *JAK2* mutation and characterised by elevated red cell mass and increased risk of thrombotic events. Patients are managed with phlebotomy to maintain haematocrit (Hct) < 0.45, and patients stratified as ‘high risk’ for thrombosis are additionally treated with cytoreductive agents to attain this target.

**Study:**

This analysis of newly diagnosed *JAK2* mutant PV patients (*n* = 50) over 2 years aimed to determine how effectively patients attained and maintained target Hct according to recommended practice.

**Conclusions:**

We found that patients spent the majority of time in target Hct range. Findings are supportive of current management guidelines.

## Background

Polycythaemia vera (PV) is a myeloproliferative neoplasm attributed to genetic variation in *JAK2* in > 97% of cases, usually by V617F and less commonly by exon 12 mutations. This leads to increased red cell mass and Hct and consequently increased risk of thrombotic events [1]. There is longer term risk of transformation to myelofibrosis or acute leukaemia, particularly in older patients. Management primarily aims to minimise thrombotic risk via antiplatelet drugs, phlebotomy, and cytoreductive agents with target Hct of 0.45 [2]. Cytoreduction is reserved for patients stratified as high risk for thrombosis [3, 4] or in selected low-risk patients [5].

Previous studies have reported concern that patients are not being adequately cytoreduced and have insufficiently stringent Hct control [6], with potential for unacceptable thrombotic consequences and a call for a change in practice. In real-world clinical practice we have little knowledge of the efficacy of cytoreduction in maintaining individual patients in range. We wished to evaluate how effectively Hct targets were being attained and maintained in this patient group based on current evidence-based guidelines.

## Methods

Lab records were used to collate all *JAK2* V617F variant cases retrospectively identified between Jan 2019 and Jan 2021, then stratified according to MPN subtype, yielding a cohort of *JAK2* V617F mutant PV patients who had been referred for testing from primary and secondary care throughout Northern Ireland. Electronic records were used to collect data pertaining to risk category as per British Society of Haematology (BSH) criteria [3], treatment (venesection, cytoreduction, or both; month at which cytoreduction started), and target Hct. Hb and Hct were recorded at 0, 3, and 6 months and 6-monthly thereafter, allowing determination of time-point at which target Hct was first attained and proportion of time-points thereafter in target range (%). Where venesection was used, newly diagnosed patients typically underwent weekly phlebotomy until in target range. As regards cytoreduction, dosage strategy was determined by the treating physician according to efficacy. For the purposes of analysis, a target Hct of 0.45 was assumed in cases where this was not documented.

A weighted ‘time in range index’ (TIRI) was calculated using the following formula to avoid skew attributable to shorter follow-up: (time-points in range − time-points out of range)/total time-points recorded. TIRI_maint_ was then calculated to reflect maintenance of time in range after target Hct attained, using only ‘post-attainment’ figures to generate the numerator value.

Information was also collected as to whether aspirin (or alternative) was recommended, cardiovascular risk factor management was recommended, and multidisciplinary team (MDT) discussion of management took place prospectively as per local practice.

## Results

Sixty-two *JAK2* V617F mutant PV patients were identified from lab records and screened in chronological order. Due to availability of complete data, a cohort of 50 patients were left for analysis. Median follow-up was 24 months (range 6–42 months). Forty-six high-risk and 4 low-risk patients were included, which was expected in this area due to patient age.

**Fig. 1:**
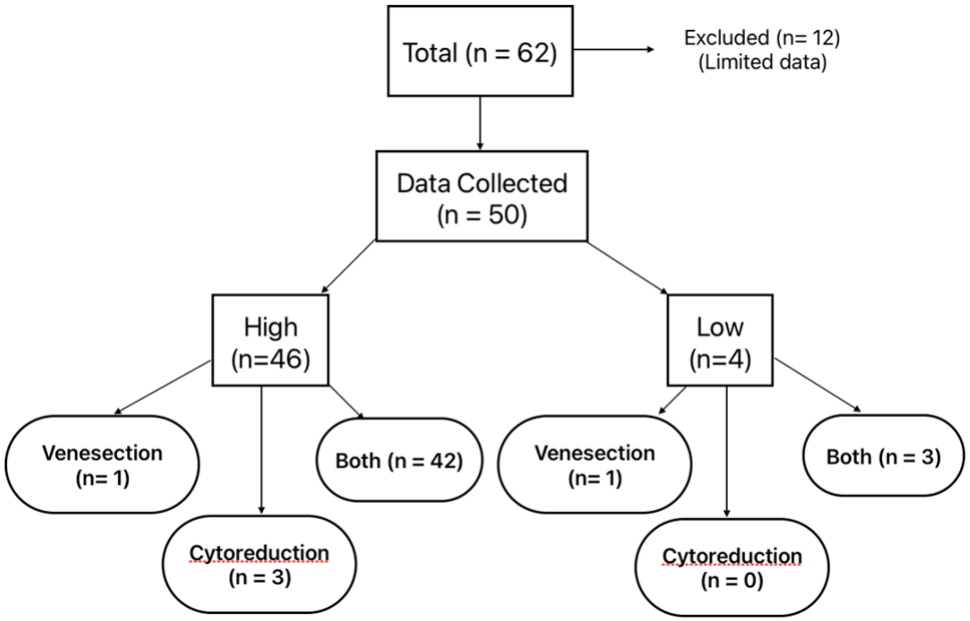
Breakdown summary of patient characteristics

One low-risk and 1 high-risk patient were managed with phlebotomy alone; this high-risk patient had declined cytoreduction. The majority (*n* = 48) were managed with either cytoreduction alone (*n* = 3, all high-risk) or in combination with phlebotomy (*n* = 45). Having reviewed the data, only small numbers of patients were noted to be on interferon and the vast majority on hydroxycarbamide, where cytoreductive agents prescribed. The indications for cytoreduction in low-risk patients (*n* = 3) were itch, splenomegaly, and failure to attain target range with phlebotomy alone; these three patients were discussed at MDT and were managed as per recent ELN guidelines [5] (Fig. 1).

**Fig. 2:**
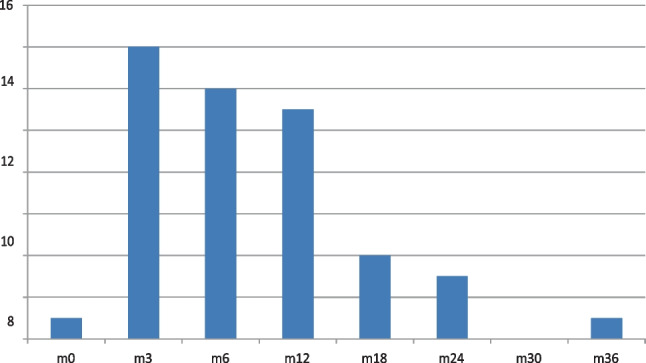
Number of high-risk patients attaining target haematocrit per month (m)

**Fig. 3:**
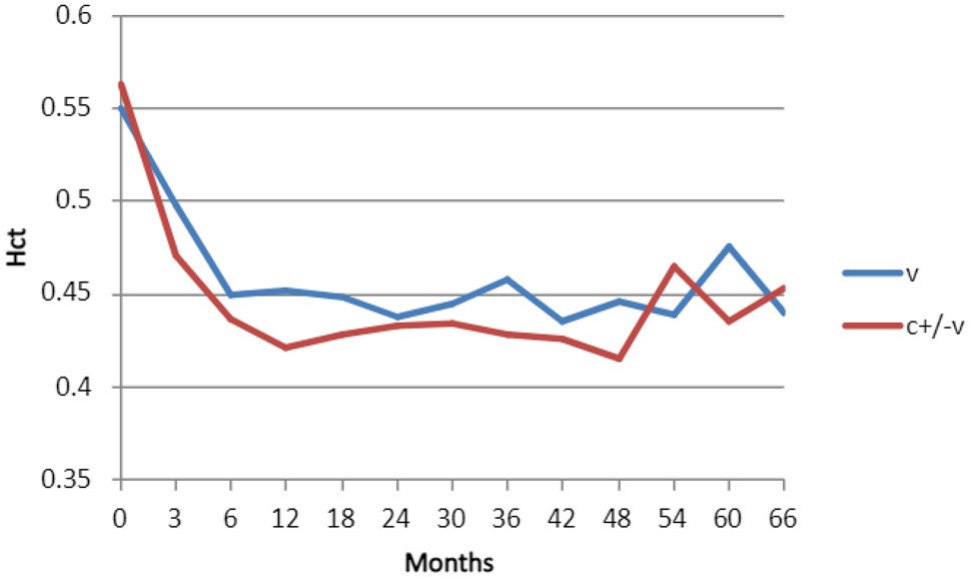
Mean haematocrit (Hct) for all patients according to treatment modality (v = venesection, c ± v = cytoreduction with or without venesection)

Most high-risk patients had attained Hct 0.45 by 6 months (Fig. 2). Four patients had not attained target Hct at 18 months. Fifty percent of patients spent > 75% of time-points in range and 70% of patients spent > 50% of time-points in range. Mean haematocrit appeared to be lower in patients receiving cytoreductive therapy (Fig. 3).

**Fig. 4:**
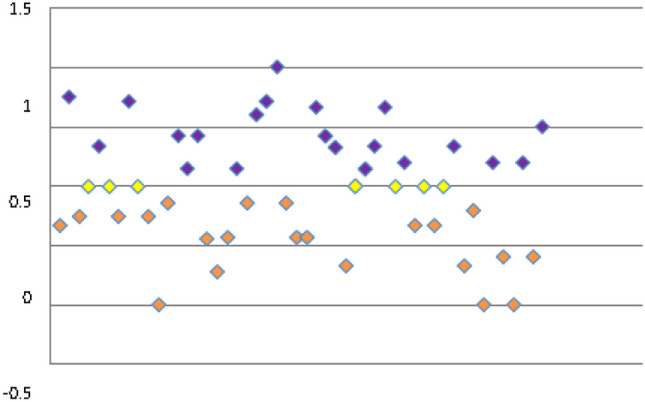
Time in range by calculated index (TIRI). The more positive the index, the more time spent in range

The mean TIRI for all patients was − 0.02 (median 0, range − 1–1; Fig. 4). When filtered for high-risk patients on cytoreduction (*n* = 45), the mean TIRI was + 0.01 (median 0, range − 1–0.75). Mean TIRI_maint_ for all patients was + 0.25 (median + 0.27, range − 0.42–1). Filtered for high-risk patients on cytoreduction, mean TIRI_maint_ was + 0.28 (median + 0.29, range − 0.43–1).

All patients were treated with aspirin or alternative antiplatelet or anticoagulant. Target haematocrit was set at 0.45 in 38/50 cases and not recorded in the remaining 12 cases. Recommendation for management of cardiovascular risk factors was recorded in 39/50 cases. MDT discussion was documented in 45/50 cases.

## Discussion

We found that there was good compliance with risk-stratified treatment guidelines in this cohort with use of cytoreduction in most patients. Patients usually attained target Hct within 6 months and subsequently tended to remain within target range. It appeared that the majority of patients spent the majority of the time with Hct in target range, once target Hct initially achieved.

Weighted analysis was carried out to account for patients with shorter follow-up, or patients taking longer to attain target range, e.g. those treated with interferon. During the studied time frame of median 2 years, TIRI and TIRI_maint_ values indicate that once diagnosed, patients will spend approximately half of time within target range. If we focus on the period after target range is first attained, patients spend more than half of time in target range. This was demonstrated for all patients and for a selected ‘high-risk cytoreduced’ group with no apparent difference in either TIRI or TIRI_maint_ between the groups; this is to be expected as the two groups are very similar.

This cohort was otherwise managed according to best practice as regards antiplatelet/anticoagulant prophylaxis, communication of target Hct, and recommendation of the need for cardiovascular risk management. It is likely that regular discussion of such cases at MDT, particularly for challenging or atypical cases, has contributed to good practice in this regard.

This study would have benefited from a longer follow-up period to generate more complete data and a more accurate and reliable TIRI_maint_ (i.e. more values included in the post-attainment/maintenance period of treatment). It seems likely that the TIRI and TIRI_maint_ would remain positive with longer follow-up but this study is unable to demonstrate this hypothesis.

Given the relatively short follow-up and relatively small cohort included, this study was not powered to assess rate of thrombotic events following diagnosis. It is likely that given the known low rate of thrombosis amongst *JAK2* variant PV patients on antiplatelet or anticoagulation prophylaxis, insignificant numbers of thrombotic events would have been noted.

Conversely, the rationale for thrombotic risk stratification in PV is such that low-risk patients can avoid potential adverse effects of cytoreductive therapies. This study did not record adverse effects of treatment and cannot advocate for a more conservative ‘phlebotomy-only’ approach in high-risk patients. However, current guidelines are based on a strong body of evidence recognising the value of cytoreduction over its limitations, in selected patients.

Hct is used as a proxy measure of red cell mass as per standard practice although it is accepted that this is not the gold standard.

Previous studies have demonstrated concern that cytoreduction was not being used sufficiently to maintain patients in target range but this is contested by the presented data [6, 7]. This study supports current UK best practice guidelines and also demonstrates the value of MDT discussion for both specific cases and communication purposes.

This study demonstrates the efficacy of current real-world management in maintaining individual patients within target range, as previous studies have demonstrated an average Hct per time-point for a study population. This study recognises individual fluctuations in Hct over time, likely due to the multiple variables affecting Hct including hydration, compliance, concomitant drug therapies, and medical conditions. We have confirmed that cytoreduction is sufficiently effective in the majority of the time and supports its use particularly in high-risk patients.
